# 619. Mpox Vaccine Uptake among Black Sexual Minority Men

**DOI:** 10.1093/ofid/ofaf695.192

**Published:** 2026-01-11

**Authors:** Samuel Opara, Sabriya L Linton, Kamini Doraivelu, McKinsey Bullock, Srija Dutta, Antonio Newman, Marcus O Reed, Daniel I Alohan, Nata M Assad, Leslie M Carson, Kyle J Moon, Tsedenia Tewodros, Brian W Weir, Sophia A Hussen

**Affiliations:** Emory University, Atlanta, GA; Bloomberg School of Public Health, Johns Hopkins University, Baltimore, Maryland; Emory University, Atlanta, GA; Emory University, Atlanta, GA; Emory University, Atlanta, GA; Emory University, Atlanta, GA; Emory University, Atlanta, GA; Emory University, Atlanta, GA; Emory University, Atlanta, GA; Bloomberg School of Public Health, Johns Hopkins University, Baltimore, Maryland; Bloomberg School of Public Health, Johns Hopkins University, Baltimore, Maryland; Emory University, Atlanta, GA; Bloomberg School of Public Health, Johns Hopkins University, Baltimore, Maryland; Emory University, Atlanta, GA

## Abstract

**Background:**

Following the global outbreak of clade II mpox in 2022 and the more recent emergence of clade I mpox in Central and Eastern Africa, mpox remains a significant public health concern both nationally and globally. During the 2022 outbreak, racial and sexual minority groups in the United States bore a disproportionate burden of disease, and mpox vaccine uptake was suboptimal among racial minority groups. In light of the ongoing low-level spread of clade II mpox, and the potential for future outbreaks, we 1) assessed the vaccination-to-case ratio for mpox, and 2) characterized factors associated with mpox vaccine uptake, in a cohort of Black sexual minority men (SMM) in Atlanta, Georgia.

**Figure:**
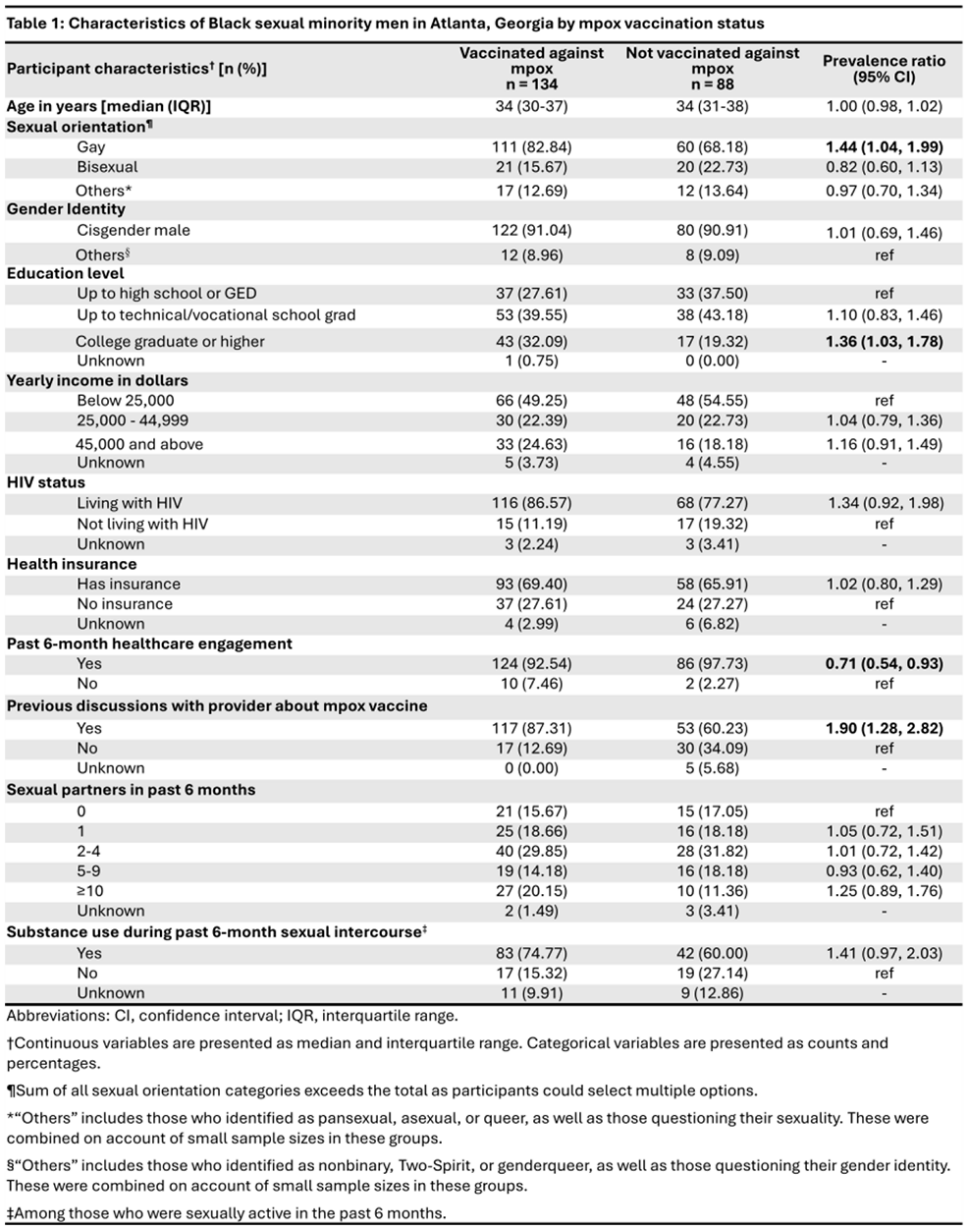

**Figure:**
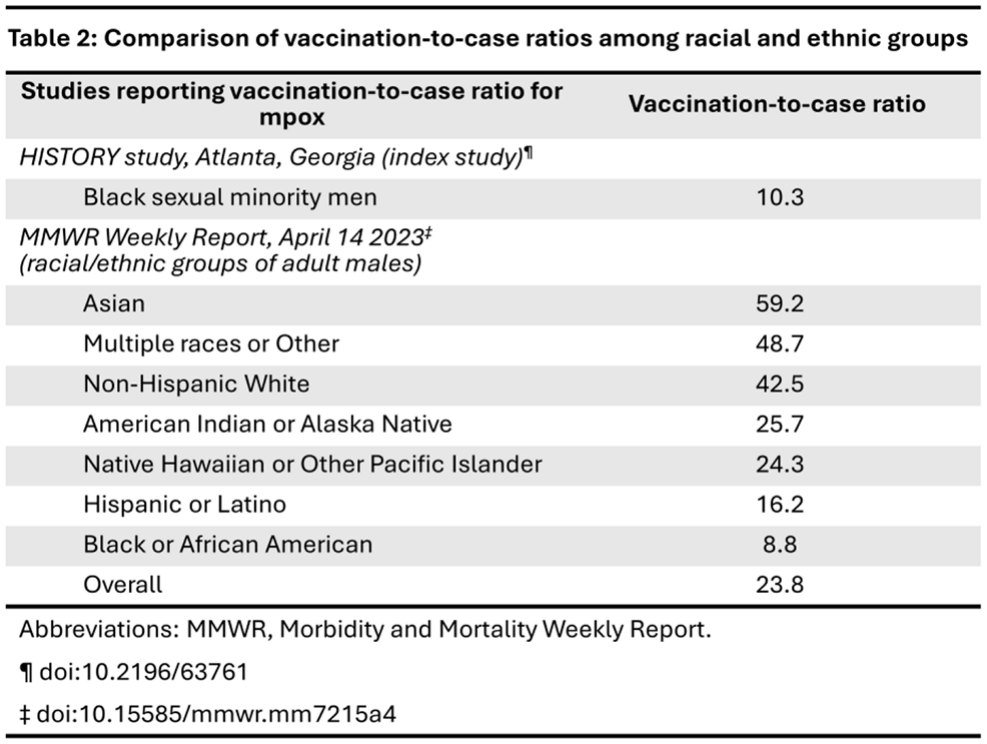

**Methods:**

We analyzed cross-sectional, self-reported data from 222 Black SMM, aged 18-44 years, enrolled in the HISTORY study in Atlanta. The vaccination-to-case ratio was calculated as the number of participants who had ever received the mpox vaccine divided by the number who reported a past-year mpox infection. We compared sociodemographic, sexual behavioral, and healthcare-related characteristics in vaccinated vs. unvaccinated participants using crude prevalence ratios (PR) and 95% confidence intervals (CI) computed via log-binomial regression.

**Results:**

Overall, 134 participants (60.36%) were vaccinated against mpox (Table 1), and 13 (5.86%) reported past-year mpox infection, with a vaccination-to-case ratio of 10.31 (Table 2). Vaccinated participants were more likely to 1) identify as gay (PR 1.44; 95% CI 1.04, 1.99), 2) be a college graduate or higher (PR 1.36; 95% CI 1.03, 1.78), and 3) report previous mpox vaccine-related discussions with a provider (PR 1.90; 95% CI 1.28, 2.82). Vaccinated participants were less likely to have engaged with healthcare in the past 6 months (PR 0.71; 95% CI 0.54, 0.93).

**Conclusion:**

Mpox vaccine uptake remains suboptimal in this high priority group of Black SMM. The vaccination-to-case ratio for this cohort is comparable to a previously reported national estimate for Black males in 2022, and lower than previously reported for all other racial groups. Discussions with healthcare providers may play a significant role in improving mpox vaccine uptake in Black SMM.

**Disclosures:**

All Authors: No reported disclosures

